# The Effect of Prostaglandin F2 Analog Treatment on the Immunoexpression of Fibrosis-Associated Factors in Patients with Glaucoma Undergoing Deep Sclerotomy

**DOI:** 10.3390/ijms252312618

**Published:** 2024-11-24

**Authors:** Robert Stanić, Katarina Vukojević, Natalija Filipović, Benjamin Benzon, Marin Ogorevc, Nenad Kunac, Samir Čanović, Petra Kovačević, Martina Paradžik Šimunović, Suzana Konjevoda

**Affiliations:** 1 Department of Ophthalmology, University Hospital in Split, Šoltanska 1, 21000 Split, Croatia; robertstanic@hotmail.com (R.S.); martina.paradzik@gmail.com (M.P.Š.); 2 Department of Anatomy, Histology and Embryology, University of Split School of Medicine, Šoltanska 2, 21000 Split, Croatia; natalija.filipovic@mefst.hr (N.F.); benjamin.benzon@mefst.hr (B.B.); marin.ogorevc@mefst.hr (M.O.); 3 Department of Pathology, Forensic Medicine and Cytology, University Hospital in Split, Spinčićeva 1, 21000 Split, Croatia; nenad.kunac@mefst.hr; 4 Department of Ophtalmology, General Hospital Zadar, Ul. Bože Peričića 5, 23000 Zadar, Croatia; canovicsamir@yahoo.com (S.Č.); suzana.konjevoda@gmail.com (S.K.); 5 Department of Ophthalmology, University Hospital Centre Zagreb, Kišpatićeva 12, 10000 Zagreb, Croatia; petra.kovacevic110@gmail.com; 6 Department of Health Studies, University of Zadar, Ulica Mihovila Pavlinovica 1, 23000 Zadar, Croatia

**Keywords:** glaucoma, prostaglandins, CTGF, HSP70, aSMA, cMYB, HIFa, SNAIL

## Abstract

Long-term use of topical prostaglandins might initiate chronic conjunctival inflammation, leading to poor outcomes of glaucoma surgery. The aim of this study was to evaluate the immunoexpression pattern of HSP70, CTGF, SNAIL, aSMA, cMYB, and HIFa in the conjunctiva, episclera, and deep sclera in patients with glaucoma undergoing deep sclerectomy in order to establish an association between staining intensities and prostaglandin F2 (PGF2) treatment. Double immunofluorescence (HSP70, CTGF, SNAIL, aSMA, cMYB, and HIFa) was performed on conjunctiva, episclera, and deep sclera samples, which were obtained from 23 patients treated with PGF2 and 8 patients without PGF2 treatment. When comparing the ocular tissues of patients regarding treatment with PGF2 analogs, we found a significant increase in the immunoexpression of HSP70 in the conjunctival epithelium of patients treated with PGF2 analogs compared to those without PGF2 treatment. These patients also had an increase in SNAIL immunoexpression and a decrease in aSMA immunoexpression in the deep sclera. There were no significant differences in HIFa, CTGF, or cMYB immunoexpression levels between the two groups. Further research into the regulation of these factors in ocular tissues could lead to the development of potential novel therapeutic approaches in glaucoma management.

## 1. Introduction

Glaucoma is one of the leading causes of blindness worldwide, and approximately 79.6 million people have glaucoma today, with a positive trend, the prevalence is expected to increase to 111.8 million in 2040 [1,2]. The main therapeutic objective of treating glaucoma is reducing intraocular pressure (IOP), which has a positive effect on slowing down the loss of visual acuity [3]. The main therapeutic approaches are medication or laser treatment, while in more severe cases resistant to therapy, the gold standard in the treatment of glaucoma is a surgical approach [4,5,6,7]. According to the European Glaucoma Society, a leading organization that provides guidelines in glaucoma management, prostaglandin drop therapy provides the highest reduction of IOP and should be considered first when initiating monotherapy treatment for glaucoma [8]. Prostaglandins act by increasing aqueous humor outflow via predominantly the uveoscleral and trabecular pathways [8,9]. Experimental evidence indicates that levels of prostaglandins are higher in the cornea/sclera than in the aqueous humor, suggesting that distribution via the conjunctival/scleral absorption route should be considered in a concentration–activity relationship [10]. As a consequence of prostaglandin proinflammatory potential, an increase in prostaglandin E2 (PGE2) and matrix metalloproteinases (MMPs) levels can cause tissue remodeling [11]. Unfortunately, long-term glaucoma therapy results in an increased incidence of conjunctival inflammation, which can negatively affect the success of the surgical operation [12,13]. Even when surgery is meticulously performed, the risk of surgical failure due to conjunctiva scarring, fibrosis, and occlusion of the new outflow pathway remains.

Scar creation in the filtering pathway subsequent to glaucoma surgery is mostly generated by the fibroblast’s proliferation and deposition of collagen. Connective tissue growth factor (CTGF) is a key player in the control of extracellular matrix remodeling, fibrosis, and angiogenesis [14]. Additionally, CTGF is associated with the process of wound healing and inflammatory response [15]. In normal tissue homeostasis, there is very low or no immunoexpression of CTGF in the area of aqueous humor outflow. As described previously, downregulation of CTGF could decrease proliferation and increase apoptosis of fibroblasts, therefore being a target for potential therapy [16]. The cMyb (c-myeloblastosis) transcription factor controls the differentiation of hematopoietic cells, where its role is best understood. Additionally, CTGF, cMyb, and Snail are also important in fibrosis and epithelial-mesenchymal transition (EMT) [17]. Snail not only functions as a key influencer of EMT but also plays a main role in the survival of cells and immune regulation. The immunoexpression of Snail positively correlates with tumor development and recurrence. Alpha smooth muscle actin (aSMA) is a marker of myofibroblast proliferation. Targeted inhibition of aSMA used in an experimental glaucoma study performed on cultured human periscleral fibroblasts showed consequent decreased fibroblast proliferation, which indicates that aSMA is a potential glaucoma therapeutic target. Heat shock protein 70 (HSP70) is a member of the family of ubiquitously expressed heat shock proteins, mainly playing a role in inducing apoptosis. Previous studies found the presence of HSP 70 in patients with glaucoma, suggesting tissue remodeling in the development of disease [18]. Hypoxia-inducible factor alpha (HIFa) is a marker of cellular response due to hypoxia, and it also induces transcription of genes involved in cell proliferation. In the rat animal model of ocular hypertension, the immunoexpression level of HIF-1α showed a significant positive correlation with IOP, suggesting a critical adaptive reaction to hypoxia in glaucoma [19].

The aim of our study was to determine whether treatment with PGF2 analogs affects the immunoexpression of fibrosis-associated factors in the conjunctiva, episclera, and deep sclera in order to predict the success of surgical outcome in patients treated with glaucoma filtering surgery.

## 2. Results

### 2.1. Hematoxylin and Eosin Staining and Mallory Staining

**Figure 1:**
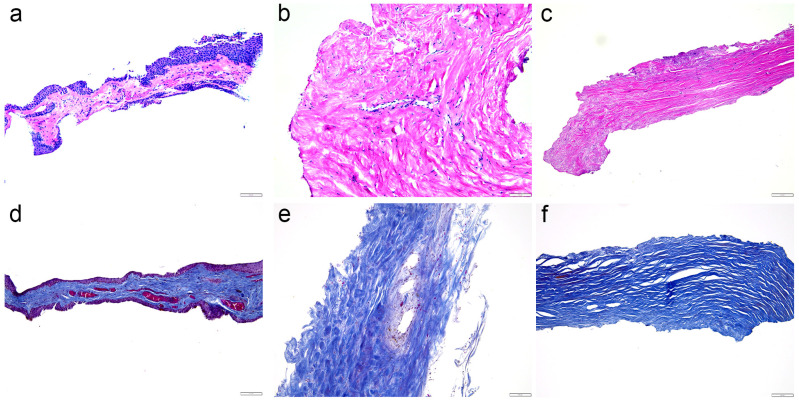
Morphology of the analyzed tissues. The figure displays hematoxylin and eosin staining (**a**–**c**) and Mallory trichrome staining (**d**–**f**) of the conjunctiva (**a**,**d**), episcleral (**b**,**e**), and deep sclera (**c**,**f**) of patients with glaucoma. These are all representative images. Scale bars represent 100 µm.

The conjunctival epithelium appeared as a stratified columnar epithelium, which in some areas transitions to stratified squamous. Above the cuboidal basal layer, the cells were more flattened and polygonal as they progressed towards the surface. The apical surface of the epithelium was smooth. Beneath the epithelial layer lies the conjunctival stroma, which is composed of loose connective tissue. This region contained scattered fibroblasts and blood vessels (Figure 1a). No anatomical variability was seen. The episclera, lying beneath the conjunctiva and above the sclera, is a denser connective tissue layer. It contained fewer cells compared to the conjunctival stroma and had a more organized, collagen-rich matrix. Fibroblasts were less frequent and had spindle-shaped nuclei. Blood vessels were prominent and often accompanied by perivascular inflammatory cells (Figure 1b). The sclera was characterized by its dense, fibrous structure, primarily composed of parallel bundles of collagen fibers. Scattered among the collagen fibers are fibroblasts with elongated, spindle-shaped nuclei. The sclera was relatively avascular compared to the other ocular tissues, but it did contain some blood vessels. The superficial sclera showed a slightly more organized collagen arrangement, while the deep sclera had a more interwoven pattern (Figure 1c).

Mallory trichrome staining effectively differentiates the various components of the conjunctiva, episclera, and sclera, emphasizing collagenous structures, cellular details, and vascular elements. The cytoplasm of the conjunctival epithelium was stained in varying shades of purple, while the nuclei were dark red. The underlying stroma stained a vibrant blue due to the collagen fibers. Fibroblast nuclei within the stroma stained dark red, standing out against the lighter background of the connective tissue. Blood vessels within the stroma were highlighted by the red staining of intravascular erythrocytes (Figure 1d). The episcleral connective tissue appeared densely packed with blue-staining collagen fibers. This dense blue staining indicates the higher collagen content and more fibrous nature of the episclera compared to the conjunctival stroma. Blood vessels were prominent, with endothelial cells and erythrocytes demonstrating red staining (Figure 1e). The scleral tissue was predominantly composed of dense collagen fibers that stained a deep blue, indicating its high collagen content. Scattered fibroblasts were present within the dense collagenous matrix, with their nuclei staining dark red. The scarce blood vessels that were present were clearly visible with endothelial cells showing dark red nuclei and red-stained erythrocytes within the lumen (Figure 1f).

### 2.2. Immunofluorescence Staining

**Figure 2:**
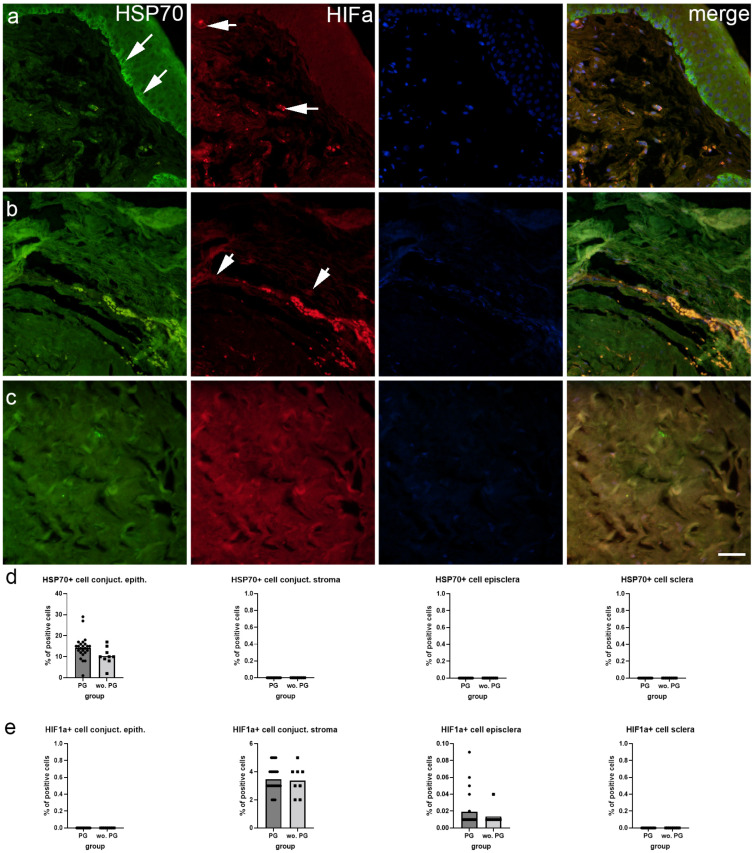
Immunoexpression of heat shock protein 70 (HSP70), hypoxia-inducible factor alpha (HIFa) and 4′,6-diamidino-2-phenylindole (DAPI) nuclear staining image and merged HSP70, HIFa, and DAPI staining in the conjunctiva (**a**), episcleral (**b**), and deep sclera (**c**) of patients with glaucoma. The arrows show the immunoexpression pattern of HSP70 and HIFa in the analyzed tissues. Images were taken at a magnification of ×400. The scale bar is 50 µm, which refers to all images. The percentage of cells positive for HSP70 (**d**) and HIFa (**e**) in the conjunctiva, episcleral, and deep sclera of patients with glaucoma is represented by bar graphs. The bars represent the mean of these data, while the circles and squares represent individual datapoints of the analyzed samples: patients with prostaglandin (PG) treatment (circles) and without PG treatment (squares). These data were analyzed using the unpaired t-test with Welch’s correction.

HSP70 immunoexpression was observed in the conjunctival epithelium, while conjunctival stroma, episclera, and deep sclera were negative (Figure 2a–c). There were significantly more HSP70-positive cells in the conjunctival epithelium of patients treated with prostaglandin F2 alpha analogs (PGF2) compared to patients without PGF2 treatment (*p* = 0.043) (Figure 2d). HIFa immunoexpression was present in the conjunctival stroma and episclera, while the deep sclera and conjunctival epithelium were devoid of any staining (Figure 2a–c). There were no significant differences between patients with or without PGF2 treatment (Figure 2e).

**Figure 3:**
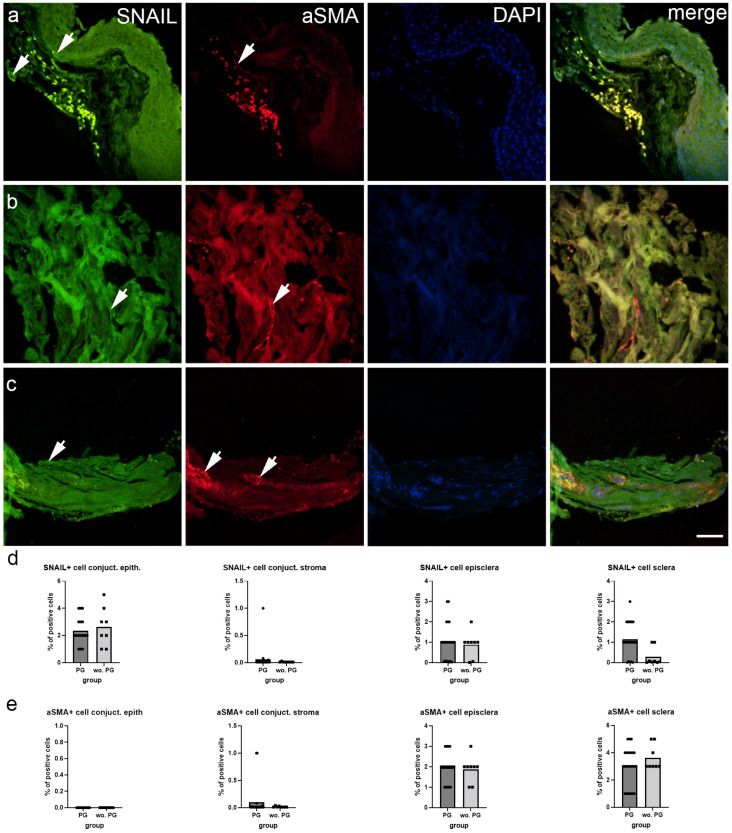
Immunoexpression of zinc finger protein SNAIL (SNAIL), alpha-smooth muscle actin (aSMA) and 4′,6-diamidino-2-phenylindole (DAPI) nuclear staining image and merged SNAIL, aSMA, and DAPI staining in the conjunctiva (**a**), episcleral (**b**), and deep sclera (**c**) of patients with glaucoma. The arrows show the immunoexpression pattern of SNAIL and aSMA in the analyzed tissues. Images were taken at a magnification of ×400. The scale bar is 50 µm, which refers to all images. The percentage of cells positive for SNAIL (**d**) and aSMA (**e**) in the conjunctiva, episcleral, and deep sclera of patients with glaucoma are represented by bar graphs. The bars represent the mean of these data, while the circles and squares represent individual datapoints of the analyzed samples: patients with prostaglandin (PG) treatment (circles) and without PG treatment (squares). These data were analyzed using the unpaired t-test with Welch’s correction.

SNAIL immunoexpression was abundant in the conjunctival epithelium and sparce in the stroma, while episclera and deep sclera showed moderate immunoexpression levels of SNAIL (Figure 3a–c). SNAIL immunoexpression was significantly higher in the deep sclera of patients treated with PGF2 compared to patients without treatment (*p* = 0.001), while there were no differences between patient groups in the other tissues (Figure 3d). There was no aSMA staining in the conjunctival epithelium and only in a few cells of the stroma. Both the episcleral and deep sclera showed visible aSMA staining (Figure 3a–c). There was a significantly higher percentage of aSMA positive in the deep sclera of patients without PGF2 treatment compared to patients treated with PGF2 (*p* = 0.050) (Figure 3e).

**Figure 4:**
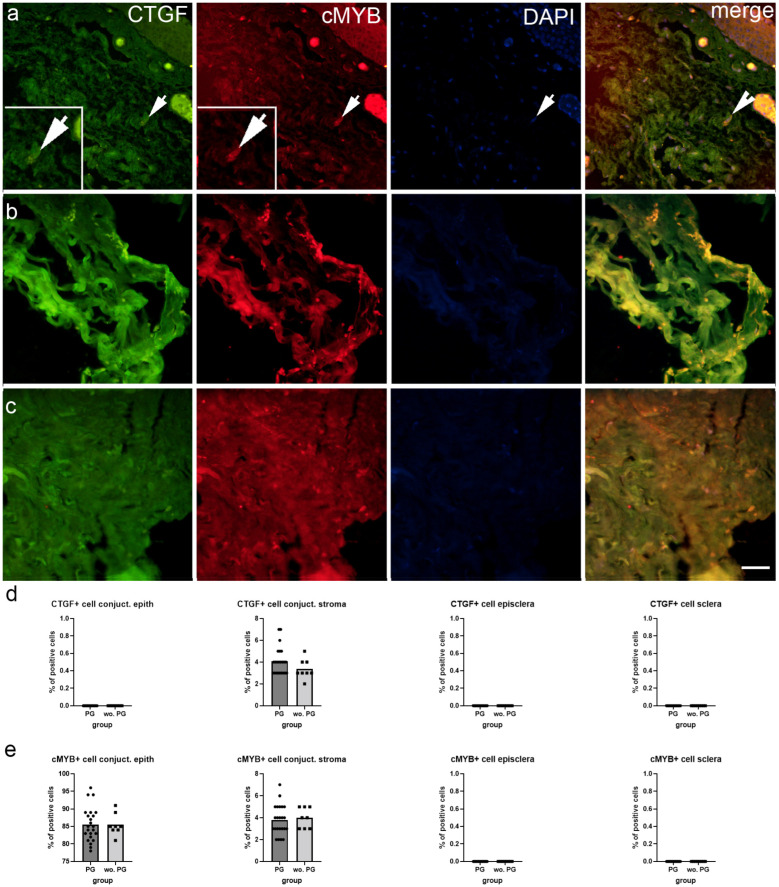
Immunoexpression of connective tissue growth factor (CTGF), transcription factor cMYB (cMYB) and 4′,6-diamidino-2-phenylindole (DAPI) nuclear staining image and merged SNAIL, aSMA, and DAPI staining in the conjunctiva (**a**), episcleral (**b**), and deep sclera (**c**) of patients with glaucoma. The arrows show the immunoexpression pattern of CTGF and cMYB in the analyzed tissues. Images were taken at a magnification of ×40. The scale bar is 50 µm, which refers to all images. The percentage of cells positive for CTGF (**d**) and cMYB (**e**) in the conjunctiva, episcleral, and deep sclera of patients with glaucoma is represented by bar graphs. The bars represent the mean of these data, while the circles and squares represent individual datapoints of the analyzed samples; patients with prostaglandin (PG) treatment (circles) and without PG treatment (squares). These data were analyzed using the unpaired t-test with Welch’s correction.

CTGF immunoexpression was observed only in the conjunctival stroma, while the epithelium, episclera, and deep sclera had no CTGF immunoexpression (Figure 4a–c). cMYB was immunoexpressed in the conjunctiva, both in the epithelium and the stroma, while the episcleral and deep sclera were negative for cMYB immunoexpression (Figure 4a–c). There were no significant differences in CTGF and cMYB immunoexpression between patients when taking into account PGF2 treatment (Figure 4d,e).

## 3. Discussion

Hematoxylin and eosin staining of our human samples revealed typical morphology of the conjunctiva, episclera, and deep sclera, which is in line with the observations in other studies [20,21,22]. Mallory trichrome staining revealed differences in the collagen density between the analyzed tissues, with the deep sclera containing the most densely packed collagen fibers, as was described previously [22].

We performed immunofluorescence staining that provided additional insights into the differential immunoexpression of various proteins across these ocular tissues, particularly in response to PGF2 treatment. HSP70 immunoexpression was prominent in the conjunctival epithelium but absent in the stroma, episclera, and deep sclera. Notably, the conjunctival epithelium of patients treated with PGF2 showed a significantly higher number of HSP70-positive cells compared to untreated patients. The conjunctival epithelium serves as a barrier against external insults and pathogens, making it susceptible to various stressors [23,24]. The upregulation of HSP70 in this region may indicate an adaptive response to protect the epithelial cells from stress-induced damage and maintain their function [25]. The observation of a significantly higher number of HSP70-positive cells in the conjunctival epithelium of patients with glaucoma treated with PGF2 compared to untreated patients suggests a potential link between PGF2 treatment and the induction of a stress response or protective mechanism mediated by HSP70. Elevated HSP70 might also indicate a stress response to therapy, potentially contributing to inflammation and fibrosis. Understanding the role of HSP70 in the ocular tissues of patients with glaucoma, particularly in response to PGF2 treatment, has implications for optimizing therapeutic strategies. Targeting HSP70 pathways could potentially enhance the protective mechanisms in the conjunctival epithelium, thereby improving ocular surface health and overall treatment outcomes in patients with glaucoma.

Our findings revealed the immunoexpression pattern of HIFa in the conjunctival stroma and episclera, while no staining was detected in the deep sclera and conjunctival epithelium. The presence of HIFa in the conjunctival stroma and episclera implies a crucial role for this transcription factor in regulating cellular responses to hypoxia in these regions [26]. In ocular tissues, especially under hypoxic conditions possibly induced by prolonged drug use or inflammation, HIFa might exacerbate fibrotic changes, making it a relevant marker to investigate in relation to prostaglandin F2 analog effects. Considering that the conjunctival stroma and episclera are highly vascularized tissues, the immunoexpression of HIFa in these regions may indicate an adaptive response to maintain cellular function and promote angiogenesis under hypoxic conditions [27,28]. Although PGF2 is commonly used in glaucoma management to reduce intraocular pressure, it does not appear to directly influence the immunoexpression of HIFa in these ocular tissues.

Our findings revealed the immunoexpression pattern of SNAIL, a key regulator of epithelial-mesenchymal transition (EMT), in various ocular tissues of patients with glaucoma. SNAIL was found to be abundant in the conjunctival epithelium, sparse in the stroma, and moderately expressed in the episclera and deep sclera. Notably, SNAIL immunoexpression was significantly higher in the deep sclera of patients treated with PGF2 compared to untreated patients. The differential immunoexpression of SNAIL across the ocular tissues suggests its involvement in regulating EMT processes in specific regions [17]. Increased immunoexpression of SNAIL would suggest that EMT is occurring in response to prostaglandin F2 analog treatment, which may exacerbate fibrotic responses in ocular tissues. The expression of SNAIL in the conjunctival epithelium indicates a potential for EMT in this region, which may be important for maintaining epithelial integrity and responding to various stimuli [29,30]. The sparse expression in the stroma suggests a lower propensity for EMT in this tissue, while the moderate expression in the episclera and deep sclera implies a more dynamic regulation of EMT processes in these regions. The significantly higher expression of SNAIL in the deep sclera of patients treated with PGF2 compared to untreated patients suggests that PGF2 treatment may influence EMT processes specifically in the sclera. The sclera, being a connective tissue layer, is known to undergo remodeling and fibrosis in various ocular diseases, including glaucoma [22]. The increased SNAIL expression in the deep sclera of patients treated with PGF2 may indicate potential tissue remodeling in response to the treatment. Understanding the role of SNAIL and EMT in the ocular tissues of glaucoma patients, particularly in response to PGF2 treatment, has implications for optimizing therapeutic strategies and managing potential side effects. The upregulation of SNAIL in the deep sclera of patients treated with PGF2 suggests the need for further investigation into the long-term effects of the treatment on scleral remodeling and fibrosis. Additionally, exploring potential interventions to modulate EMT processes in the sclera may lead to the development of novel therapeutic approaches to prevent or mitigate scleral remodeling and fibrosis in patients with glaucoma.

Our findings provide insights into the expression pattern of aSMA staining, a marker for myofibroblast activity [31], in various ocular tissues of patients with glaucoma. The results indicate that aSMA staining was absent in the conjunctival epithelium, minimal in the stroma, visible in the episclera, and present in the deep sclera. The differential expression of aSMA staining across the ocular tissues reflects the distribution of myofibroblast activity in patients with glaucoma, and it was previously demonstrated on a mouse model that glaucoma is associated with increased scleral fibroblast proliferation and myofibroblast differentiation [32]. We found a significantly higher percentage of aSMA-positive cells in the deep sclera of untreated patients compared to those treated with PGF2, which implies that PGF2 treatment may reduce myofibroblast activity or differentiation in the deep sclera. This is in contrast with previous findings, which determined that PGF2 promotes lung and myocardial fibrosis by stimulating fibroblast proliferation [33]. Namely, increased aSMA expression would indicate the presence of active myofibroblasts, suggesting that Prostaglandin F2 analog treatment might be promoting fibrosis by encouraging myofibroblast activation in the eye tissues. Additionally, the study by Kalouche et al. determined that treatment with the PGF2 analog latanoprost promotes aSMA expression and myofibroblast transition in human trabecular meshwork cells [34]. Considering the opposing findings about the effect of PGF2 on aSMA expression in our and other studies, additional studies are needed to better understand their connection. The observed reduction in aSMA-positive cells in the deep sclera of patients treated with PGF2 highlights the potential of PGF2 to mitigate myofibroblast-mediated fibrotic processes in the sclera, which could contribute to improved treatment outcomes in glaucoma.

Our findings provide insights into the expression patterns of CTGF and cMYB in various ocular tissues of patients with glaucoma. The results indicate that CTGF expression was restricted to the conjunctival stroma, while cMYB was expressed in both the conjunctival epithelium and stroma, with no detectable expression in the episclera and deep sclera. Notably, there were no significant differences in CTGF and cMYB expression between PGF2-treated and untreated patients. The differential expression of CTGF and cMYB across the ocular tissues suggests distinct roles for these factors in maintaining tissue homeostasis and responding to various stimuli. CTGF, a key regulator of fibrosis and tissue remodeling [35], was specifically expressed in the conjunctival stroma, implying a potential role in modulating the stromal microenvironment. In patients with glaucoma receiving Prostaglandin F2 analogs, CTGF could mediate fibrosis in the conjunctiva, potentially affecting the surgical outcome of deep sclerotomy by promoting excessive scarring. cMYB, a transcription factor involved in cell proliferation and differentiation [36], was expressed in both the conjunctival epithelium and stroma, suggesting a broader role in regulating cellular processes in these tissues. High levels of c-MYB could indicate enhanced cell proliferation in the sclera and conjunctiva, which might result in increased tissue thickness and rigidity post-surgery. The absence of significant differences in CTGF and cMYB expression between PGF2-treated and untreated patients indicates that PGF2 treatment does not markedly alter the expression of these factors in the conjunctival and scleral tissues.

## 4. Materials and Methods

### 4.1. Study Population

The study design was reviewed and approved by the Ethics Committee of the General Hospital Zadar (Zadar, Croatia). Patients received information about this study, and after obtaining informed consent, they were recruited to deep sclerotomy procedure. Inclusion criteria were as follows: (1) Age ≥ 18 years (2) Diagnosis of primary or pseudo-exfoliation glaucoma (3) Minimum of 2 years administration of topical prostaglandin antiglaucoma drug. Exclusion criteria were as follows: (1) All previous ocular surgery. In this study, we enrolled 23 patients treated with PGF2 and 8 patients without PGF2 treatment. Surgery process started with opening conjunctiva and Tenon’s capsule at the limbus in upper quadrant. Exposed scleral surface was abraded with Hockey knife (Alcon Ophthalmic Kit, Fort Worth, TX, USA), and bleeding episcleral veins were cauterized. A superficial scleral flap sizing 5 × 5 mm was created with 11 surgical blades (British AILEE brand) following deep scleral flap sizing 4 × 4 mm that was constructed with 15 surgical knives (Ophthalmic Kit, Fort Worth, TX, USA). Both flaps were finished with crescent blades (Ophthalmic Kit, Fort Worth, TX, USA). Succeeding the exposure of trabeculodescement membrane and peeling off the inner wall of Schlemm’s canal, a deep scleral flap was excised and analyzed. Superficial flap was sealed with two 10-0 sutures (Ethylon, Ethicon, Johnson & Johnson, Bridgewater, NJ, USA), and tissue remaining between two sutures sizing 1 × 3 mm was excised and analyzed as superficial sclera sample. The conjunctiva was closed with running 8-0 sutures (Vicryl, Ethicon, Bridgewater, Johnson & Johnson, NJ, USA), and sample of limbus overriding conjunctiva was excised. An antimetabolite injection of 5-florouracil was administered into the subconjunctival tissue at the end of procedure.

### 4.2. Tissue Collection and Basic Staining Procedures

The tissue samples were collected in the period of 2011. to 2012. Samples were taken from three positions conjunctiva, episclera, and deep sclera. Samples were put into formalin solution and sent to the Department of Pathology, Forensic Medicine, and Cytology, University Hospital in Split. Tissues were paraffin-embedded, and then all other tissue procedures and histological analyses were performed at the Department of Anatomy, Histology, and Embryology of the University of Split School of Medicine. Paraffin-embedded tissues were cut into 5 μm thick sections. Each 10th section was stained with hematoxylin and eosin to check appropriate tissue quality for immunofluorescence staining.

### 4.3. Double Immunofluorescence

After deparaffinization, sections for immunohistochemistry were rehydrated in decreased ethanol grades as we described previously [37,38,39]. Briefly, the slides were heated in citrate buffer (pH 6.0) in a microwave oven for 10 min. After cooling off, the slides were rinsed with PBS. Block protein was then put on the tissue sections for 30 min following overnight incubation with appropriate primary antibody mixture (Table 1). On the next day, the slides were rinsed with PBS and an appropriate combination of secondary antibodies was applied (Table 1).

The nuclei were stained with 4′,6-diamidino-2-phenylindole (DAPI), and slides were mounted in Immumount and coverslipped. Slides were examined using an Olympus (Tokyo, Japan) BX51 microscope equipped with a Nikon DS-Ri1 camera (Nikon Corporation, Tokyo, Japan). Images were assembled using Adobe Photoshop (Adobe Systems, MI, USA). Five non-overlapping fields were taken from each sample from each position using 40× objective magnification. Double immunofluorescence with combination of primary antibodies: CTGF and cMyb; Snail and aSMA; HSP70 and HIF1a was used (Table 1) to determine the number of positive cells in each position of the conjunctiva, episclera, and deep sclera. To eliminate the background signal “levels” tool in Adobe Photoshop (v21.0.2, Adobe, San Jose, CA, USA), it was applied only for representative images assembled in Figure 2 and Figure 4. All image processing for analysis and quantification was performed using (v1.530 ImageJ, NIH, Bethesda, MD, USA). Briefly, the green signal was isolated by removing the red color channel. After duplicating the images, on one of them, the median filter was applied and subtracted from the unfiltered image to identify the positive signal. By using the “triangle” option, the final photos were thresholded, converted to 8-bit images, and merged with DAPI. Cell count was performed using ImageJ software 1.53m (National Institutes of Health, Bethesda, MD, USA). The total number of positive cells was calculated as number of cells per mm2 in conjunctiva, episclera, and deep sclera. The final total number per patient was the mean of 10 sections that were calculated and compared between groups.

### 4.4. Statistical Analysis

Data analysis was conducted with GraphPad Prism (GraphPad Software 8.0, La Jolla, CA, USA). The normality of data distribution was determined by the Kolmogorov–Smirnov test. The unpaired t-test with Welch’s correction was used for statistical analysis to examine the difference between tissues of patients with and without PGF2 analog treatment. Statistical significance was set at *p* < 0.05.

## 5. Conclusions

The patients’ clinical data and immunofluorescence analyses of the observed factors provide a comprehensive understanding of the structural and functional variations across the analyzed ocular tissues. The differential expression of HSP70, SNAIL, and aSMA in response to PGF2 treatment highlights the complex interplay between stress response mechanisms, EMT, myofibroblast differentiation, and therapeutic interventions in ocular diseases. Additionally, our study highlights an important aspect of glaucoma management by comparing two surgical techniques: trabeculotomy and deep sclerotomy. Given the conjunctiva’s susceptibility to inflammation following antiglaucoma drug therapy, our findings suggest that the deeper drainage route provided by deep sclerotomy can help reduce postoperative complications. By creating an intrascleral drainage path, deep sclerotomy minimizes dependence on the conjunctiva, which can be more vulnerable after prolonged medical therapy. This insight is significant for glaucoma surgeons as it proposes a strategic shift toward procedures that lower intraocular pressure with potentially fewer postoperative complications. These findings could encourage a preference for deeper drainage surgeries, especially in patients with extensive histories of topical therapy, as these patients often have conjunctival inflammation that may compromise surgical outcomes if procedures like trabeculotomy are performed. Although our study contributes valuable evidence to the ongoing improvement of surgical interventions in glaucoma care, further research into the specific mechanisms underlying the regulation of different factors used and their impact on ocular health is needed for better glaucoma management.

## Figures and Tables

**Table 1:** Primary and secondary antibodies.

Antibodies	Catalog Number	Host	Dilution	Source
Anti-HSP70	ab31010	Rabbit	1:100	Abcam (Cambridge, UK)
HIF1a	sc-13515	Mouse	1:200	Santa Cruz Bt. (Dallas, TX, USA)
Anti-Snail	ab53519	Goat	1:500	Abcam (Cambridge, UK)
Anti-Smooth Muscle Actin	M0851	Mouse	1:300	Dako, Glostrup, Denmark
CTGF	sc-14939	Goat	1:200	Santa Cruz Bt. (Dallas, TX, USA)
c-Myb	sc-7874	Rabbit	1:200	Santa Cruz Bt. (Dallas, TX, USA)
Alexa Fluor^®^488 AffiniPure Anti-Goat lgG (H+L)	705-545-003	Donkey	1:400	Jackson Immuno Research Laboratories, Inc. Baltimore, PA, USA
Anti-Rabbit IgG, Alexa Fluor^®^ 488,	711-545-152	Donkey	1:400	Jackson Immuno Research Laboratories, Inc. (Baltimore, PA, USA)
Rhodamine Red™-X (RRX) AffiniPure Anti-Mouse IgG (H+L)	715-295-151	Donkey	1:400	Jackson Immuno Research Laboratories, Inc. Baltimore, PA, USA
Anti-Rabbit IgG, Alexa Fluor^®^ 488,	711-545-152	Donkey	1:400	Jackson Immuno Research Laboratories, Inc. (Baltimore, PA, USA)
Rhodamine Red™-X (RRX) AffiniPure Donkey Anti-Rabbit IgG (H+L)	711-295-152	Donkey	1:400	Jackson Immuno Research Laboratories, Inc. I (Baltimore, PA, USA)

## Data Availability

All data and materials are available upon request due to GDPR restrictions.
